# Kuijieling-Containing Serum Regulates Th17 and Treg Cell Differentiation by Inhibiting STAT3 Signaling *In Vitro*

**DOI:** 10.1155/2019/7837989

**Published:** 2019-08-25

**Authors:** Yu Long, Yuqing He, Fengming Jie, Sixin Li, Yanli Wu, Yanwu Li, Qun Du

**Affiliations:** Pi-Wei Institute, Guangzhou University of Chinese Medicine, Guangzhou, Guangdong 510006, China

## Abstract

**Object:**

To investigate the effect of Kuijieling (KJL) on the balance between T helper 17 (Th17) and regulatory T (Treg) cells in peripheral blood mononuclear cells (PBMC) *in vitro* and explore the underlying mechanism.

**Materials and Methods:**

PBMCs isolated from rats were stimulated with transforming growth factor-*β*, interleukin (IL)-6, and IL-23 to induce the imbalance of Th17 and Treg cells and were treated with 10, 5, or 2.5% KJL-containing serum. The proportion of Th17 or Treg cells in CD4^+^ T cells was analyzed by flow cytometry, the concentrations of IL-17, IL-21, and IL-10 were assayed by ELISA, mRNA expressions of retinoic acid-related orphan receptor *γ*t (ROR*γ*t), forkhead box protein 3 (Foxp3), and signal transducer and activator of transcription 3 (STAT3) were quantified by PCR, and phosphorylated STAT3 (p-STAT3) was analyzed by flow cytometry.

**Results:**

KJL-containing serum decreased the proportion of Th17 cells and increased the proportion of Treg cells in CD4^+^ T cells, decreased the concentration of IL-17 and IL-21, enhanced the level of IL-10 in the cell culture supernatant, promoted the expression of Foxp3, and inhibited the levels of ROR*γ*t, STAT3, and p-STAT3.

**Conclusion:**

KJL suppresses the STAT3 pathway to remedy the imbalance between Th17 and Treg cells.

## 1. Introduction

Ulcerative colitis (UC) is an idiopathic chronic inflammation in the colon mucosa [1]. The incidence rate of UC in developed countries has increased markedly in the last century [2]. Chinese medicine may have an effect on UC and could be considered as an adjunct therapy [3]. Chinese medicine theory considers that heat induced by blood stasis supports the manifestation of UC and that deficiency of the spleen is the intrinsic basis of this condition [4]. Kuijieling (KJL) is a Chinese medicine formula used at Guangzhou University of Chinese Medicine, Guangzhou, China, to treat UC, considered to reinforce the spleen, dissipate heat, and activate the blood [5]. The KJL formula contains Ilicis Rotundae Cortex (Jiu Bi Ying, bark of Ilex rotunda Thunb.), Rhizoma Atractylodes Macrocephalae (Bai Zhu, root and stem of *Atractylodes macrocephala* Koidz.), Radix Paeoniae Alba (Bai Shao, root of *Paeonia lactiflora* Pall.), Hirudo (Shui Zhi, *Whitmania pigra* Whitman), and Radix Glycyrrhizae Preparata (Zhi Gan Cao, fried root of *Glycyrrhiza uralensis* Fisch.). Previous studies demonstrated that KJL ameliorates symptoms in patients with UC and in an experimental colitis rat model [6, 7]. In a recent study exploring its mechanism, we found that KJL regulates certain associated cytokines and transcription factors to inhibit T helper 17 (Th17) cells and promote regulatory T (Treg) cells in rats with colitis [8].

A recent study demonstrated that Th17 and Treg cells are involved in UC [9]. Increased numbers of Th17 cells and decreased numbers of Treg cells were detected in the peripheral blood of patients suffering from UC, and the imbalance of these cell types contributes to inflammation in this disease [10–12]. Th17 cell is a subset of helper T cells characterized by the production of interleukin (IL)-17 [13]. Retinoic acid-related orphan receptor *γ*t (ROR*γ*t) is the transcription factor marker of Th17 cells, the equivalent of T-box transcription factor TBX21 in Th1 cells and trans-acting T-cell-specific transcription factor GATA-3 in Th2 cells [14]. Th17 cells benefit epithelium barrier function and serve to defend against potential pathogens in the healthy physiological state [15]. However, in pathological conditions, these cells secrete excessive IL-17, recruit neutrophils and monocytes, increase the production of TNF-*α* and IL-1*β*, and aggravate autoimmune inflammation [16]. Th17 cells contribute to the occurrence of the majority of autoimmune diseases, including UC [17, 18]. The Treg cells are a subtype of T cells that perform anti-inflammatory function and maintain immune homeostasis [19]. Forkhead box protein 3 (Foxp3) is the transcription factor marker of Treg cells [20]. Cell-cell interactions and the secretion of IL-10 are the main mechanisms used by Treg cells to regulate the immune response [19]. A lack of these cells can induce autoimmune inflammation [21]. According to the opposite function, a theory about the balance between Th17 and Treg cells has been put forward for autoimmune and inflammatory diseases [22]. Therapies targeting the equilibrium between these two cell types are potential therapeutic approaches for UC [14].

Signal transducer and activator of transcription 3 (STAT3) is a deciding factor in Th17 and Treg cell differentiation [23]. The cytokines that are critical for the development and maintenance of Th17 cells, including IL-6, IL-21, and IL-23, all function primarily through STAT3 signaling [24–26]. The differentiation of Th17 cells and production of IL-17 are halted in the absence of STAT3 [23, 27]. On the other hand, phosphorylated STAT3 (p-STAT3) inhibits the development of Treg cells and the production of IL-10 [23]. Aberrant STAT3 signaling induces the imbalance of Th17 and Treg cells [23].

|The effect of KJL on the equilibrium between Th17 and Treg cells on the molecular level remains unknown. In the present study, we constructed a cell model of the imbalance between Th17 and Treg cells *in vitro*, evaluated the effect of KJL-containing serum on this model, and further revealed the role of the STAT3 signaling pathway, thereby demonstrating the mechanism of the effect of KJL on UC.

## 2. Materials and Methods

### 2.1. Preparation of KJL and KJL-Containing Serum

The five components of KJL, including Ilicis Rotundae Cortex (Jiu Bi Ying, bark of Ilex rotunda Thunb.), Rhizoma Atractylodes Macrocephalae (Bai Zhu, root and stem of *Atractylodes macrocephala* Koidz.), Paeoniae Radix Alba (Bai Shao, root of *Paeonia lactiflora* Pall.), Hirudo (Shui Zhi, *Whitmania pigra* Whitman), and Radix Glycyrrhizae Preparata (Zhi Gan Cao, fried root of *Glycyrrhiza uralensis* Fisch.), in the proportion of 4 : 2 : 2 : 2 : 1, were purchased from Caizhilin chain-store (Guangzhou, China). The herbs were verified by Jiayun Tong (Teaching and Research Section for Authentication of Chinese Medicine, Guangzhou University of Chinese Medicine) as meeting the standards in pharmacopoeia of China. After soaking in distilled water for 30 min, all the herbal components were decocted in distilled water for 1 h. The decoction was concentrated using a water bath, to a final concentration of 1.83 g herbal mix per ml. Male Sprague Dawley (SD) rats were divided into a KJL group and a control group. The rats in the KJL group were administered 10 ml/kg KJL by gavage for two days. The rats were then fasted for 24 h and administered KJL two more times with an interval of 2 h. The animals in the control group were given the same volume of water at the same time points. Blood was collected from the abdominal aorta 1 h after the last administration, left to clot for 30 min, and centrifuged at 3,000 rpm for 15 min to isolate the serum. The collected serum was sterilized using a 0.22 *μ*m membrane filter, heated with 50°C water for 30 min, and stored at −20°C. Serum from the KJL group was labelled KJL-containing serum, and that from the control group was the control serum.

### 2.2. Cell Isolation

Male SD rats (weight, 180–220 g) were anesthetized with chloral hydrate, and blood was collected from the abdominal aorta using vacuum blood collection tubes with heparin in sterile conditions. Peripheral blood mononuclear cells (PBMCs) were isolated by Ficoll-Hypaque density gradient centrifugation (TBD, China). A volume of 5 ml blood was layered onto 5 ml Ficoll-Hypaque and centrifuged at 2,500 rpm for 25 min. The PBMC layer was collected and washed with PBS twice.

### 2.3. Cell Viability Assay

Isolated PBMCs were divided into four groups and cultured with different media at 1 × 10^6^ cells/ml: 10% control serum + 90% RPMI-1640 medium for the control group; 10% KJL-containing serum + 90% RPMI-1640 medium for the high KJL dose (KDH) group; 5% KJL-containing serum + 5% control serum + 90% RPMI-1640 medium for the medium KJL dose (KDM) group; and 2.5% KJL-containing serum + 7.5% control serum + 90% RPMI-1640 medium for the low KJL dose (KDL) group. Rat anti-CD3 mAb (BioLegend, Inc., USA) and rat anti-CD28 mAb (BD Pharmingen, USA) were used for activation of the T cells in the PBMCs. The 96-well culture plates for PBMC culture were precoated with 5 *μ*g/ml anti-CD3 mAb at 4°C overnight. Anti-CD28 mAb was added into the medium at a concentration of 2 *μ*g/*μ*l. The cell viability at 12, 24, and 48 h was analyzed using an MTT Cell Proliferation and Cytotoxicity Assay kit (Beijing Solarbio Science & Technology Co., Ltd., China).

### 2.4. Cell Culture

Isolated PBMCs were divided into six groups and cultured in different media at 1 × 10^6^ cells/ml: 10% control serum + 90% RPMI-1640 medium for the control, model, and hydrocortisone (HC) groups; 10% KJL containing serum + 90% RPMI-1640 medium for the KDH group; 5% KJL containing serum + 5% control serum + 90% RPMI-1640 medium for the KDM group; and 2.5% KJL containing serum + 7.5% control serum + 90% RPMI-1640 medium for the KDL group. Rat anti-CD3 mAb (BioLegend, Inc., USA) and rat anti-CD28 mAb (BD Pharmingen, USA) were used for activation of the T cell in the PBMCs. The 24-well culture plates for PBMC culture were precoated with 5 *μ*g/ml anti-CD3 mAb at 4°C overnight. Anti-CD28 mAb was added into the medium at a final concentration of 2 *μ*g/*μ*l. Polarization towards Th17 cells was induced with 5 ng/ml transforming growth factor-*β* (TGF-*β*), 20 ng/ml IL-6, and 25 ng/ml IL-23 (all PeproTech, Inc., USA) in all groups except for the control group, to cause an imbalance between Th17 and Treg cells. For the HC group, 20 ng/ml HC was added into the medium. All groups of cells were cultured under conditions of 5% CO_2_ and 37°C for 48 h prior to subsequent experiments.

### 2.5. Flow Cytometry Analysis of the Proportion of Treg and Th17 Cells in CD4^+^ T Cells

Each group was divided into two. One subgroup was stained for the quantification of Treg cells. The cells were washed twice with PBS, resuspended in 100 *μ*l PBS, and incubated with rat anti-CD4 FITC and rat anti-CD25 APC (both eBioscience; Thermo Fisher Scientific, Inc., USA) at 4°C in the dark for 15 min. After the extracellular staining, the cells were washed twice with PBS, fixed, and permeabilized using the Foxp3 Staining Buffer set (eBioscience; Thermo Fisher Scientific, Inc., USA) at 4°C in the dark for 50 min. Following the fixation and permeabilization, the cells were washed once with permeabilization buffer (eBioscience; Thermo Fisher Scientific, Inc., USA), resuspended in 100 ml permeabilization buffer, and incubated with anti-mouse/rat Foxp3 PE (eBioscience; Thermo Fisher Scientific, Inc., USA) at 4°C in the dark for 30 min. Equal amount of rat IgG2a K isotype control PE (eBioscience; Thermo Fisher Scientific, Inc., USA) was used for normalization and to confirm antibody specificity. After the intracellular staining, the cells were washed twice with permeabilization buffer, resuspended in 200 *μ*l 4% paraformaldehyde solution, and prepared for analysis. The second subgroup of cells was prepared for Th17 cell analysis. The cells were stimulated using a cell-stimulation cocktail (eBioscience; Thermo Fisher Scientific, Inc., USA) for 6 h before staining. The staining steps for Th17 cell analysis were similar to those for Treg analysis, except the extracellular staining was performed with rat anti-CD4 FITC and the intracellular staining with anti-mouse/rat IL-17A PE (eBioscience; Thermo Fisher Scientific, Inc., USA). The samples were analyzed on a BD Accuri™ C6 flow cytometer. CD4^+^CD25^+^Foxp3^+^ cells were recognized as the Treg cells and CD4^+^IL-17^+^ cells as the Th17 cells.

### 2.6. Measurement of IL-17, IL-21, and IL-10 Concentrations in the Cell Culture Supernatant by ELISA

The cell suspension was centrifuged at 1,000 rpm for 5 min to isolate the supernatant. The supernatant collected was centrifuged at 7,000 rpm for 5 min to remove residual cells and was stored at −80°C. The concentrations of IL-17, IL-21, and IL-10 in the supernatant were measured by specific ELISA kits (Cusabio Technology, LLC, China), following the manufacturer's protocols.

### 2.7. ROR*γ*t, Foxp3, and STAT3 mRNA Quantification by Reverse Transcription-Quantitative PCR (RT-qPCR)

RT-qPCR was used to measure the mRNA expression levels of ROR*γ*t, Foxp3, and STAT3. Total RNA from cells was isolated using the MiniBEST Universal RNA Extraction kit (Takara Bio, Inc., Japan), according to the manufacturer's protocol. RNA (1 *μ*g) was reverse transcribed in a 20 *μ*l reaction mixture using PrimeScript RT Master Mix (Takara Bio, Inc., Japan). After reverse transcription, 2 *μ*l cDNA was mixed with 1 *μ*l forward primer, 1 *μ*l reverse primer, 12.5 *μ*l SYBR® Premix Ex Taq II (Takara Bio, Inc., Japan), and 8.5 *μ*l RNase-free water, and the reactions were carried out in a CFX96 (Bio-Rad Laboratories, Inc., USA). The primer sequence of each target mRNA is as follows: 5′-GCTGCTGACCCCAGTATGAA-3′ and 5′-AAAACCCCTCCCTCCCTCT-3′ for ROR*γ*t; 5′-TGAGCTGGCTGCAATTCTGG-3′ and 5′-ATCTAGCTGCTCTGCATGAGGTGA-3′ for Foxp3; and 5′-TTTGAGACAGAGGTGTACCACCAAG-3′ and 5′-ACCACAGGATTGATGCCCAAG-3′ for STAT3. The thermocycling conditions were as follows: 95°C for 30 sec and 40 cycles of 95°C for 5 sec and 60°C for 30 sec. The 2^−ΔΔCq^ method was used to quantify relative mRNA expression.

### 2.8. Determination of p-STAT3 Levels by Flow Cytometry

The expression of p-STAT3 was measured by flow cytometry. The cells were washed with PBS, fixed, and permeabilized using Foxp3 Staining Buffer set (eBioscience; Thermo Fisher Scientific, Inc., USA) at 4°C in the dark for 50 min. After the fixation and permeabilization, the cells were washed with permeabilization buffer, resuspended in 100 *μ*l permeabilization buffer, and incubated with p-STAT3 (Tyr705; D3A7) XP rabbit mAb (Cell Signaling Technology, Inc., USA) at 4°C in the dark for 1 h. After binding with the primary antibody, the cells were washed with permeabilization buffer, resuspended in 100 *μ*l permeabilization buffer, and incubated with the secondary antibody (CWBio, China) at 4°C in the dark for 1 h. Finally, the cells were washed twice with permeabilization buffer, resuspended in 200 *μ*l 4% paraformaldehyde solution, and then analyzed with a BD Accuri C6 flow cytometer. A sample incubated with equal amount of secondary antibody but not bound with the primary antibody was set as a negative control. The median fluorescence intensity was analyzed in order to evaluate the expression level.

### 2.9. Statistical Analysis

All data are displayed as mean ± standard deviation and were analyzed using SPSS version 17.0 (SPSS Inc., USA). One-way ANOVA was used for comparisons among groups.

## 3. Results

### 3.1. Effect of KJL on Cell Viability

**Figure 1:**
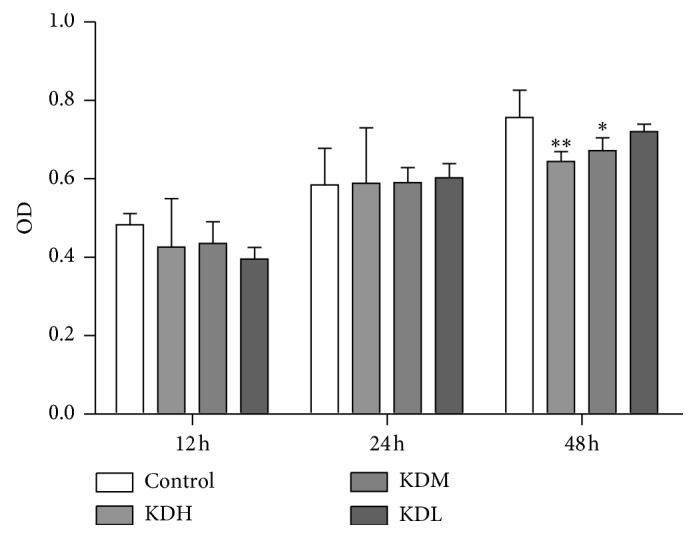
Comparison of the optical density (OD) in MTT assay among groups at 12, 24, and 48 h as measured by an MTT assay. The data are presented as the mean ± SD. ^*∗∗*^*P* < 0.01, ^*∗*^*P* < 0.05 versus the control group (*n* = 3).

The activation and proliferation of T cells in PBMCs were induced with anti-CD3 and anti-CD28. The effect of KJL on cell viability is presented in Figure 1. No significant difference was noted between each amount of KJL-containing serum and the control group at 12 and 24 h; however, at 48 h, cell viability in the KDH and KDM groups was decreased (*P* < 0.01 or *P* < 0.05).

### 3.2. KJL Leads to a Decrease in Th17 Cells and an Increase in Treg Cells

**Figure 2:**
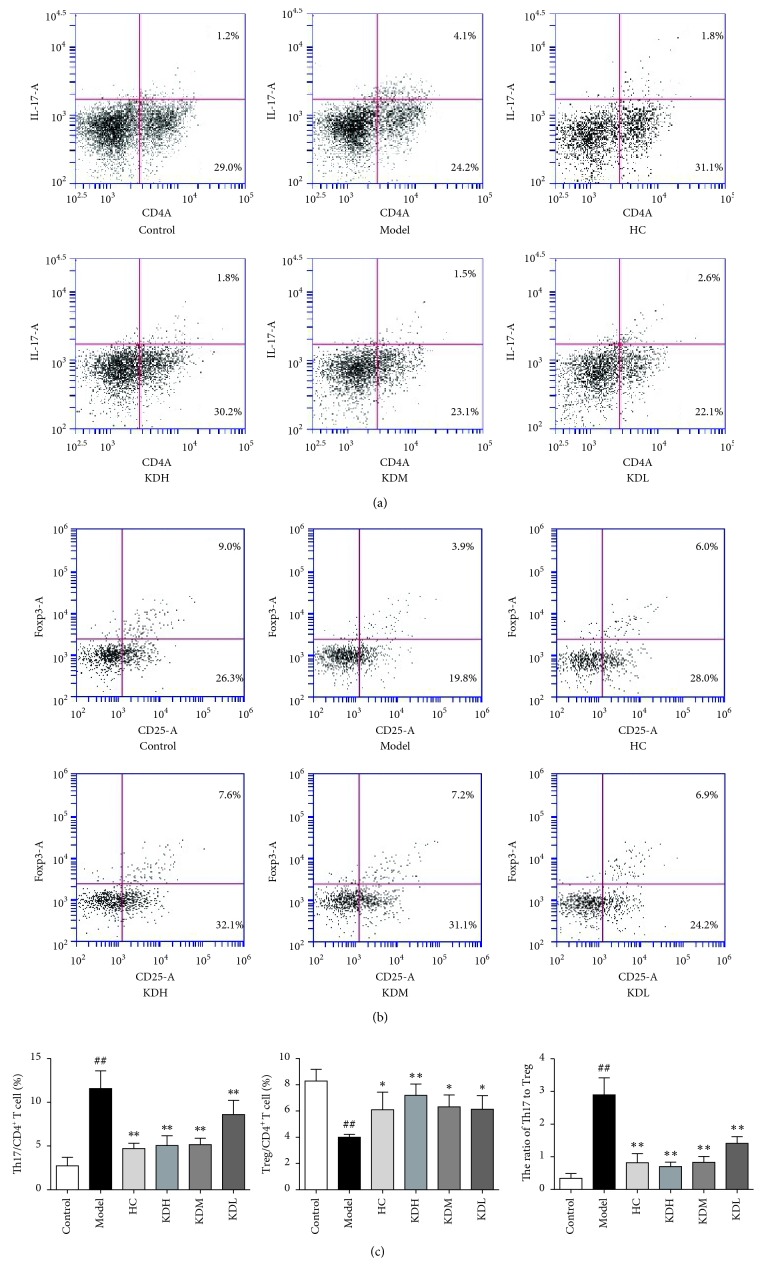
(a) The expression of CD4 and IL-17 in lymphocyte. CD4^+^IL-17^+^ cells in the pictures are Th17 cells. (b) The expression of CD25 and Foxp3 in CD4^+^ T cells. CD25^+^Foxp3^+^ in the pictures are Treg cells. (c) Comparison of the proportion of Th17 and Treg cells in CD4^+^ T cells among groups. The data are presented as the mean ± SD. ^##^*P* < 0.01 versus the control group; ^*∗∗*^*P* < 0.01, ^*∗*^*P* < 0.05 versus the model group (*n* = 4).

As presented in Figure 2, the number of Th17 cells increased (*P* < 0.01) and the number of Treg cells decreased (*P* < 0.01) in the model group compared with the control group. Administration of HC or different KJL-containing serum doses decreased Th17 cells (*P* < 0.01) and increased Treg cells (*P* < 0.01 or *P* < 0.05).

### 3.3. KJL Inhibits the Production of IL-17 and IL-21 and Promotes the Secretion of IL-10

**Figure 3:**
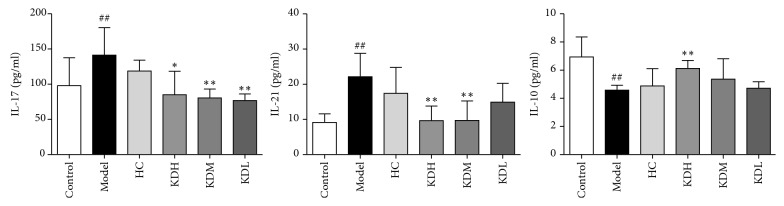
Comparison of IL-17, IL-21, and IL-10 levels in the supernatant among groups. The data are presented as the mean ± SD. ^##^*P* < 0.01 versus the control group; ^*∗∗*^*P* < 0.01, ^*∗*^*P* < 0.05 versus the model group (*n* = 3).

The concentrations of IL-17, IL-21, and IL-10 in the cell culture supernatant measured by ELISA are displayed in Figure 3. Higher levels of IL-17 and IL-21 and lower levels of IL-10 were detected in the model group compared with the control group (*P* < 0.01). Compared with the model group, IL-17 levels in the KDH, KDM, and KDL groups were decreased (*P* < 0.01 or *P* < 0.05), IL-21 levels in the KDH and KDM groups were decreased (*P* < 0.01), and IL-10 levels in the KDH group were increased (*P* < 0.01).

### 3.4. KJL Decreases the Expression of ROR*γ*t and Enhances the Level of Foxp3

**Figure 4:**
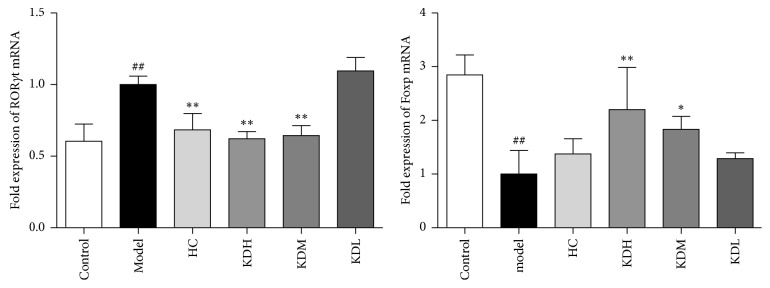
Comparison of ROR*γ*t and Foxp3 mRNA expression levels among groups. The data are presented as the mean ± SD. ^##^*P* < 0.01 versus the control group; ^*∗∗*^*P* < 0.01, ^*∗*^*P* < 0.05 versus the model group (*n* = 3).

Expression levels of ROR*γ*t and Foxp3 mRNA are shown in Figure 4. Compared with the control group, the model group exhibited increased levels of ROR*γ*t and decreased levels of Foxp3 (both *P* < 0.01). Compared with the levels in the model group, ROR*γ*t mRNA in the HC, KDH, and KDM groups were lower (all *P* < 0.01), whereas Foxp3 mRNA in the KDH and KDM groups was increased (*P* < 0.01 and *P* < 0.05, respectively).

### 3.5. KJL Decreases STAT3 and p-STAT3 Levels

**Figure 5:**
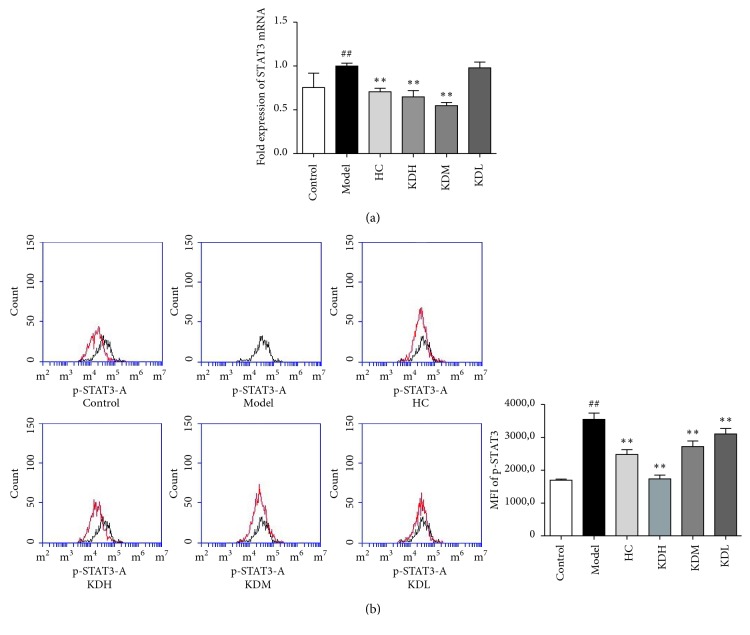
(a) Comparison of STAT3 mRNA levels among groups. (b) The expression of p-STAT3 in lymphocytes measured by flow cytometry, indicated group (red trace) versus model group (black trace), and comparison of the MFI among groups. The data are presented as the mean ± SD. ^##^*P* < 0.01 versus the control group; ^*∗∗*^*P* < 0.01, ^*∗*^*P* < 0.05 versus the model group (*n* = 3).

Expression of STAT3 mRNA, quantified by PCR, and p-STAT3 levels, measured by flow cytometry, are displayed in Figure 5. STAT3 mRNA and p-STAT3 in the model group were increased compared with the control group (*P* < 0.05). Compared with those in the model group, the levels of STAT3 mRNA and p-STAT3 in the HC, KDH, and KDM groups were decreased (all *P* < 0.01).

## 4. Discussion

Th17 and Treg cells play opposing roles in the immune response [28]. IL-17, secreted by Th17 cells, recruits neutrophils and monocytes, increasing the production of TNF-*α* and IL-1*β* and aggravating the inflammation [16]. However, IL-10, secreted by Treg cells, targets various leukocytes and represses excessive inflammatory response [21, 29]. According to the opposite function, they share a related, but antagonistic, differentiation pathway [22, 28]. TGF-*β* is the necessary cytokine for CD4^+^-naïve T cells to develop into Treg or Th17 cells. Excess TGF-*β* induces CD4^+^-naïve T cells to become Treg cells, but when TGF-*β* functions with IL-6, the cells become Th17 cells [22]. Treg cells can produce TGF-*β* and promote the generation of more Treg cells, whereas Th17 cells produce IL-21, promoting the generation of more Th17 cells [22]. Furthermore, plasticity exists in the development of Th17 or Treg cells. Their epigenetic modifications can be reprogrammed, resulting in their conversion to other subtypes [30]. Follow these discoveries, a theory about the balance between Th17 and Treg cells was proposed, regarding the pathology of autoimmune and inflammatory disease [22].

An increase in the ratio of Th17/Treg cells contributes to inflammation in UC [12]. Elevated levels of IL-6, IL-23, and IL-17 have been found in serum and colon mucosa of patients with UC, IL-23 exacerbates colon inflammation, and blocking IL-23 or IL-21 ameliorates colitis in mice, demonstrating that Th17 cells are key effector cells in UC [13, 15, 18, 31]. TGF-*β*1 and IL-10, two main effector cytokines of Treg cells, ameliorate colon inflammation in UC [32]. Deficiency of TGF-*β*1 or Foxp3, factors necessary for Treg cell differentiation, induces chronic colitis [33, 34]. These findings demonstrate that Treg cells protect against inflammation in UC. Decreased numbers of Treg cells in peripheral blood and an altered balance between Treg and effector T cells in the intestine were detected in patients with UC, indicating that the reduction of Treg cells plays a crucial role in the development of this disease [21]. The imbalance of Th17 and Treg cells is crucial in the pathophysiology of UC.

KJL is considered to be an effective therapy for UC. The effect of KJL on symptoms and colon pathology is similar to that of sulfasalazine [6]. When KJL was used in combination with sulfasalazine, fewer adverse effects and a markedly enhanced curative rate were observed [6]. In a colitis rat model, the number of ulcers, pathological scale, and TNF-*α*, IL-1*β*, and IL-6 levels were reduced following treatment with KJL [7, 35]. The value and potential of this mixture on the treatment of UC are notable, but the mechanism remains unknown. In a previous study, we found that KJL decreases Th17 cells and increases Treg cells in rats with colitis [36]. Subsequently, we identified that KJL enhances the expression level of Foxp3 and reduces ROR*γ*t levels in these animals, thereby regulating Treg and Th17 cell differentiation, promoting the production of IL-10, and inhibiting the secretion of IL-17 and IL-21 [8]. We also found that KJL decreases STAT3 protein expression in colon mucosa, indicating that the STAT3 signaling pathway may be involved [8].

In the present study, we applied serum pharmacology to explore the effect of KJL on T cell differentiation. After the compound mixture was administered to the experimental animals, their blood serum was isolated and the formula-containing serum was used in cell experiments. Using drug-containing serum is considered to be more accurate than using the crude drug when exploring the effect of Chinese medicine formulae, because many components of these treatments do not work until they have undergone a series of chemical transformations following digestion and absorption in the gastrointestinal tract [37, 38]. We activated isolated PBMCs by anti-CD3 and anti-CD28 antibodies to induce the proliferation of T cells. We found that cell proliferation was inhibited significantly by KJL-containing serum after 48 h. Increased numbers of Treg cells inhibit the effect of T cell activation and proliferation [19]; hence, the 48 h time point was chosen as the time point to observe other effects. We stimulated isolated PBMC with TGF-*β*, IL-6, and IL-23, thereby successfully establishing an imbalance of Th17 and Treg cells *in vitro*. In accordance with the animal experiments, KJL-containing serum decreased Th17 cells, increased Treg cells, and regulated the balance of Th17 and Treg cells *in vitro* [36]. We quantified the cytokines produced by the Th17 and Treg cells in order to detect the cellular function. As expected, KJL inhibited Th17 cells, decreasing IL-17 and IL-21 levels, and promoted Treg cells, increasing IL-10 levels. The key transcription factors of Th17 and Treg cells were also analyzed. Expression of ROR*γ*t was decreased, and that of Foxp3 was increased after treatment with KJL-containing serum, demonstrating that this treatment regulates the differentiation of the two cell types. Our results demonstrated the effect of KJL on Treg and Th17 cell differentiation at a molecular level.

We further revealed the role of STAT3 signaling in the effect of KJL on Th17 and Treg cells. IL-6 and IL-21 are upstream of STAT3 in the signaling cascade, so that the phosphorylation level of STAT3 is enhanced in the model of imbalance of Th17 and Treg cells [25]. The STAT3 gene is activated by STAT3 itself, and its mRNA is increased following the phosphorylation of the protein [39]. STAT3 phosphorylation promotes ROR*γ*t expression and inhibits Foxp3 expression, resulting in Th17 cell differentiation being promoted and Treg cell differentiation being inhibited in the model [23]. After treatment with KJL-containing serum, p-STAT3 and STAT3 mRNA were decreased, indicated that KJL suppresses STAT3 signaling to regulate Th17 and Treg cell differentiation. A previous study demonstrated that KJL reduces STAT3 levels in colon mucosa, where epithelial cells are the predominant cell type [8]. The present study clarified that KJL inhibits STAT3 signaling in lymphocytes, thereby regulating the balance of Th17 and Treg cells.

The results of the present study did not clarify whether the effect of KJL was the inhibition of STAT3 phosphorylation, downregulating STAT3, or the inhibition of STAT3 transcription, decreasing p-STAT3. When T cells are predominantly Th17, p-STAT3 induces STAT3 transcription. However, as a Chinese medicine composed of multiple ingredients, KJL may suppress STAT3 signaling in various ways. Certain components of KJL may contribute to the effect on the STAT3 signaling pathway. Paeoniflorin, a main active ingredient in Paeonia, suppresses STAT3 phosphorylation and promotes STAT3 degradation [40, 41]. Atractylenolide II in Atractylodes has exhibited inhibitory effects on STAT3 phosphorylation in other cells [42]. Glycyrrhizic acid from *Glycyrrhiza* decreased STAT3 and inhibited STAT3 phosphorylation via AKT/mTOR signaling [43, 44]. Our study further provided indications of the effect of these ingredients on the STAT3 signaling pathway in T cells.

In summary, our study demonstrated the effect of KJL on the differentiation of Th17 and Treg cells in PBMCs *in vitro*, revealing that KJL inhibited the STAT3 pathway to repress the polarization towards Th17 cells and promoting the polarization towards Treg cells. Our study further clarified the mechanism of KJL in treating UC and provided additional evidence supporting the application of KJL in the clinic.

## 5. Conclusion

The results of the present study showed that KJL suppresses the STAT3 pathway, thereby inhibiting Th17 cell differentiation, increasing Treg cell differentiation, and correcting the imbalance between Th17 and Treg cells.

## Data Availability

The data used to support the findings of this study are available from the corresponding author upon request.

## Abbreviations

UC: Ulcerative colitis
KJL: Kuijieling
Th17 cells: T helper 17 cells
Treg cells: Regulatory T cells
IL: Interleukin
ROR*γ*t: Retinoic acid-related orphan receptor *γ*t
Foxp3: Forkhead box protein 3
STAT3: Signal transducer and activator of transcription 3
p-STAT3: Phosphorylated signal transducer and activator of transcription
PBMCs: Peripheral blood mononuclear cells
TGF-*β*: Transforming growth factor-*β*.
